# *ALDH1L2* orchestrates redox–growth coupling in renal carcinoma: pan-cancer evidence and mechanistic validation of the ROS–Akt/mTOR/S6K axis

**DOI:** 10.3389/fimmu.2026.1768010

**Published:** 2026-02-12

**Authors:** Chao Jiang, Songsong Liu, Liwen Zhang, Shiji Li, Jinyou Wang, Yi Wang

**Affiliations:** Department of Urology, The Second Affiliated Hospital of Anhui Medical University, Hefei, China

**Keywords:** *ALDH1L2*, bladder cancer, kidney renal clear cell carcinoma, pan-cancer, prostate adenocarcinoma, RNA modification, tumor immune microenvironment

## Abstract

**Background:**

Aldehyde dehydrogenase family 1 member L2 (*ALDH1L2*) has been relatively understudied in cancer. We aimed to systematically characterize its expression patterns, clinical significance, and potential functions across cancers and to validate its biological roles in urologic tumors.

**Methods:**

Leveraging The Cancer Genome Atlas pan-cancer resource, we profiled *ALDH1L2* across tumor types with respect to expression patterns, clinical outcomes, genomic features, immune contexture, epigenetic associations, and indices of stemness and heterogeneity. Protein-level differences were examined by immunohistochemistry in bladder cancer (BLCA), prostate adenocarcinoma (PRAD), and kidney renal clear cell carcinoma (KIRC) tissues. To functionally interrogate *ALDH1L2*, we performed siRNA-mediated knockdown in relevant cell models and evaluated proliferation and motility-related phenotypes using wound-healing, Transwell, and EdU incorporation assays. In KIRC, Western blotting together with reactive oxygen species (ROS) detection was conducted to probe potential mechanistic links.

**Results:**

*ALDH1L2* was differentially expressed in multiple cancers and significantly associated with overall and disease-specific survival in KIRC. IHC showed higher ALDH1L2 expression in KIRC tissues than in adjacent normal tissues, but lower expression in BLCA and PRAD. Functionally, *ALDH1L2* knockdown suppressed proliferation and migration in KIRC cells, while promoting these processes in BLCA and PRAD cells. In KIRC, *ALDH1L2* silencing increased ROS levels and reduced Akt/mTOR/S6K phosphorylation, consistent with decreased EdU incorporation.

**Conclusion:**

This study is the first to systematically untangle the divergent roles of *ALDH1L2* in KIRC, BLCA, and PRAD from a pan-cancer perspective combined with ex vivo experiments, suggesting that *ALDH1L2* may serve as an important molecule influencing tumor progression and the immune microenvironment, thereby providing a new potential target for the diagnosis and treatment of related cancers.

## Introduction

*ALDH1L2* is a mitochondrial folate-dependent enzyme encoded at chromosome 12q23.3 (1, 2). First identified in 2010, *ALDH1L2* participates in the folate cycle by catalyzing the conversion of 10-formyltetrahydrofolate to CO2 and tetrahydrofolate, generating mitochondrial NADPH in the process, which is crucial for maintaining cellular redox balance and antioxidant defense (3). Beyond folate metabolism, emerging evidence suggests that *ALDH1L2* may influence lipid metabolism and energy homeostasis (4). For instance, *ALDH1L2* knock-out mice exhibit lipid droplet accumulation in mitochondria and reduced ATP levels, potentially linked to impaired coenzyme A biosynthesis (4). These findings indicate that *ALDH1L2* is not merely a metabolic enzyme but may also function as a regulatory factor in cellular homeostasis.

The involvement of *ALDH1L2* in tumor biology has only recently begun to be appreciated (5–7). Previous studies reported that knocking down *ALDH1L2* could inhibit distant metastasis of melanoma cells by suppressing the folate pathway (5). In clinical cohorts, elevated *ALDH1L2* expression correlated with poorer overall survival in patients with colorectal cancer and lung adenocarcinoma (1). Aging-related metabolic remodeling and redox imbalance are increasingly recognized as shared drivers of chronic diseases, including cancer, providing a rationale to interrogate mitochondrial metabolic enzymes in tumor progression (8). Given the close links between one-carbon metabolism and aging as well as circadian rhythms—both known risk factors for various malignancies (9–12)—*ALDH1L2* might represent a key metabolic regulator connecting these biological processes to tumorigenesis.

The role of *ALDH1L2* in pan-cancer remains poorly defined, which is attributed to its inconsistent functions across different cancer types. High *ALDH1L2* expression correlates with poor prognosis in pancreatic ductal adenocarcinoma (13). This enzyme is also upregulated in human colorectal tumor tissues relative to normal tissues (14), and patients with low *ALDH1L2* expression exhibit radioresistance (1). Knockdown of *ALDH1L2* has been demonstrated to suppress distant metastasis in human melanoma cells (1). Conversely, *ALDH1L2* loss can promote metastatic progression in breast cancer cells by increasing the production of formate and formylmethionine (fMet) (7). Additionally, it has been reported that *ALDH1L2* promotes hepatocellular carcinoma (HCC) progression through tumor-associated macrophage polarization, and *ALDH1L2* knockdown enhances the anti-HCC efficacy of sorafenib (15). However, a systematic pan-cancer analysis of *ALDH1L2* is currently lacking, and its context-dependent roles across different tumor types are poorly understood. Furthermore, most existing studies rely on bioinformatics analyses, lacking direct experimental validation of *ALDH1L2* function in cancer cells. Recent global analyses show that the rising burden of urinary tract tumor disease and increasing cross-border inequalities highlight the urgent need for clinically viable biomarkers and targetable pathways for urological cancers (16).

To address these gaps, we performed an integrated analysis of *ALDH1L2* across multiple human cancers using TCGA dataset. We focused on KIRC, BLCA, and PRAD, where *ALDH1L2* exhibited differential expression and prognostic significance. In addition to informatics analysis, we conducted functional experiments in cancer cell lines—including wound healing, Transwell assays, Western blotting, EdU assay, and ROS detection—to experimentally validate the role of *ALDH1L2*. Our findings provide new insights into the tumor type-specific functions of *ALDH1L2*, particularly revealing its regulatory role in the Akt/mTOR signaling pathway and ROS generation in KIRC, thereby highlighting its potential as a prognostic biomarker and therapeutic target.

## Methods

### Identification and prognostic analysis

Transcriptome profiles and matched clinical annotations for TCGA pan-cancer cohorts were obtained from the UCSC Xena browser (17–19), and *ALDH1L2* expression values were extracted for each sample. Samples from primary solid tumors, normal solid tissues, primary blood-derived cancers (TCGA-LAML), and metastatic lesions (TCGA-SKCM) were included at the data acquisition stage. To improve data quality, cases with *ALDH1L2* expression equal to zero or follow-up shorter than 30 days were removed before analysis. Expression values were then converted to log_2_(*x* + 0.001). Cancer types with fewer than ten eligible samples were removed, yielding 38 tumor entities with available *ALDH1L2* expression and survival information. Associations between *ALDH1L2* expression and survival endpoints were examined using Cox proportional hazards models, and statistical significance was assessed with log-rank tests. For tumor–normal comparisons, we considered primary tumor samples together with normal solid tissues and, where available, blood-derived normal samples. Cancer types with fewer than three normal samples, or with zero expression in all normal tissues, were excluded, resulting in 18 tumor types with usable tumor–normal information. Differences in *ALDH1L2* expression between tumor and normal tissues were evaluated using the Wilcoxon rank-sum test or the Wilcoxon signed-rank test as appropriate. Official TCGA abbreviations for each cohort included in this study are summarized in **Additional File 1: Table S1**.

### Tumor stemness, heterogeneity, and mutational landscape

We next explored whether *ALDH1L2* is linked to stem-like features in tumors. Specifically, Spearman’s rank correlation was used to relate *ALDH1L2* mRNA abundance to multiple established stemness indices spanning methylation and transcriptome-derived scores (DMPss, DNAss, ENHss, EREG.EXPss, EREG-METHss, and RNAss) (20). In parallel, a panel of heterogeneity and genome-instability measures—HRD (homologous recombination deficiency), LOH (loss of heterozygosity), NEO, tumor ploidy, tumor purity, MATH (mutant-allele tumor heterogeneity), MSI (microsatellite instability), and TMB (tumor mutational burden)—was assembled, where TMB was computed from MuTect2 mutation calls and processed with “maftools” (21, 22). After integrating mutation and expression matrices, samples carrying only synonymous alterations were excluded. Within each tumor entity, cases were dichotomized by the median *ALDH1L2* expression, and group-wise differences in mutation prevalence were evaluated using a Chi-square framework.

### RNA modification-related genes and tumor immune microenvironment

To explore potential links between *ALDH1L2* and epitranscriptomic regulation, we assessed Spearman correlations between *ALDH1L2* and 44 genes involved in RNA modifications, including writers, readers, and erasers for m1A, m5C, and m6A. Correlations between *ALDH1L2* mRNA expression and 36 inhibitory checkpoints (23), 22 stimulatory checkpoints (24), and 68 immunomodulator genes (chemokines, receptors, MHC molecules, immunoinhibitors, immunostimulators) (25) were also investigated. The TIMER and ESTIMATE algorithms were used via the R package “IOBR” (26) to evaluate the TME. Relationships between *ALDH1L2* and DNA methylation of its own locus, its mRNA expression, and tumor-infiltrating lymphocytes (TILs) (27) were further explored using the TISIDB database.

### Single-cell validation using TISCH2

To further validate the cellular distribution of *ALDH1L2* within the tumor microenvironment, we interrogated publicly available single-cell RNA-seq datasets using the Tumor Immune Single Cell Hub 2 (TISCH2) database. The “Gene” module was used to visualize *ALDH1L2* expression across annotated cell populations in urological tumor–related datasets, including BLCA, PRAD, and kidney cancer cohorts. Average expression levels were summarized by cell type as provided by the TISCH2 standardized annotation pipeline and presented as log(TPM/10 + 1).

### Tumor purity–adjusted immune correlation analysis using TIMER3

To examine whether the association between *ALDH1L2* and immune infiltration was confounded by tumor purity, we performed purity-adjusted correlation analyses using TIMER3. The “Immune–Gene” module was applied to evaluate the association between macrophage subset expression and estimated infiltration levels and CD8^+^ T cell signatures in the TCGA cohort *ALDH1L2* including BLCA, KICH, KIRC, KIRP, PRD, and TGCT. Immunoinfiltration was inferred by multiple deconvolution methods such as TIMER, EPIC, xCell, CIBERSORT/CIBERSORT-ABS, quanTIseq, MCP-counter, and Consensus-TME, and partial Spearman correlations were reported and tumor purity adjusted. A two-sided P < 0.05 was considered statistically significant.

### Immunohistochemistry and scoring

This study utilized tissue microarrays (containing bladder cancer, prostate adenocarcinoma, clear cell renal cell carcinoma tumor tissues, and corresponding adjacent tissues; purchased from Shanghai Zhuoli Biotech Company), which had passed ethical review. After routine dewaxing and rehydration of 4 μm FFPE sections, antigen retrieval was performed using heat-mediated method with citrate buffer (pH 6.0). Endogenous peroxidase activity was blocked with 3% H_2_O_2_. The sections were incubated with the primary antibody anti-ALDH1L2 (Proteintech, Rabbit, 21391-1-AP, 1:1500) at 4 °C overnight or at 37 °C for 60 mins. After washing, an HRP-conjugated secondary antibody was applied, DAB was used as the chromogen, and nuclei were counterstained with hematoxylin before dehydration and mounting. The HRP-conjugated secondary antibody and DAB chromogen were supplied in the immunohistochemistry kit (zsbio, PV-6000). The primary outcome measure was the percentage of ALDH1L2-positive area (% positive area), calculated as (DAB-positive pixel area/ROI tissue area) × 100%.

### Cell culture and transfection

Human cell models of KIRC, BLCA, and PRAD were maintained in DMEM containing 10% fetal bovine serum in a humidified incubator (37 °C, 5% CO_2_). *ALDH1L2* was knocked down using targeted siRNA. Transfection efficiency was verified by Western blot (WB).

### Wound healing assay

Cells were seeded in 6-well plates and cultured to ~90% confluence. A straight scratch was generated using a sterile 200-µL pipette tip held perpendicular to the plate surface. Detached cells were removed by gently washing twice with PBS, followed by incubation in fresh medium (with reduced serum when indicated). Images were acquired at 0 h and 24 h under identical microscope settings, and the same wound area was recorded by referencing pre-marked positions on the plate underside. Wound closure was quantified using ImageJ by measuring the wound area at each time point. The migration rate was calculated as: Wound closure (%) = (A0 − At)/A0 × 100, where A0 and At represent wound area at 0 h and time t, respectively.

### Transwell assay

Cell migration and invasion abilities were detected. Cells were fixed, stained, and counted.

### Western blotting

Cell lysates were prepared in RIPA buffer supplemented with protease and phosphatase inhibitors. Protein concentrations were quantified using a BCA assay, and 20–40 μg of total protein per sample was resolved by SDS–PAGE before transfer onto PVDF membranes. Membranes were blocked in 5% BSA (for phospho-proteins) or 5% non-fat milk and then incubated at 4 °C overnight with primary antibodies against *ALDH1L2* (Proteintech, 21391-1-AP, 1:1500), Akt (CST, 9272, 1:1000), p-Akt(Ser473)(CST, 9271, 1:1000), mTOR (CST, 2972, 1:1000), p-mTOR (Ser2448)(CST, 2971, 1:1000), p70S6K(Thr389) (Affinity, AF6226, 1:1000), p-p70S6K (Affinity, AF3228, 1:1000) and β-actin (Affinity, AF7018, 1:10000). After washing, membranes were incubated with HRP-linked secondary antibodies for 1 h at room temperature, and signals were developed using an ECL substrate. Band intensities were quantified in ImageJ; phosphorylated proteins were normalized to their corresponding total proteins and to the loading control. All assays were performed in ≥3 independent biological replicates.

### ROS detection

Intracellular ROS were assessed using DCFH-DA (Beyotime, S0033). After transfection, cells were incubated with 10 μM DCFH-DA in serum-free medium at 37 °C for 20 min in the dark, followed by three washes with PBS to remove excess probe. Fluorescence images were captured using the same exposure settings. ROS levels were quantified by ImageJ as mean fluorescence intensity and normalized to cell number, reported as relative fluorescence intensity (RFI) per 10³ cells. At least five random fields were analyzed per condition, and experiments were repeated in three independent biological replicates.

### EdU assay

DNA synthesis and proliferative activity were assessed using an EdU incorporation kit (Beyotime, ST067).Cells were exposed to EdU for a defined labeling period, fixed, permeabilized, and subjected to a click-chemistry reaction to fluorescently label incorporated EdU, while nuclei were counterstained with DAPI. Images were acquired using fluorescence microscopy. The EdU labeling index was quantified using ImageJ as the percentage of EdU-positive nuclei among total DAPI-stained nuclei:EdU labeling index (%) = (EdU^+^nuclei/total nuclei) × 100. At least five random fields were quantified per condition, and experiments were repeated in three independent biological replicates.

### Statistical analysis

All statistical analyses were performed using R software (version 3.6.4) and appropriate R packages.

Non-parametric tests, including the unpaired Wilcoxon rank-sum test and the Wilcoxon signed-rank test, were used for two-group comparisons, while the Kruskal–Wallis test was applied for multiple-group comparisons. Unless otherwise specified, all *p* values were two-sided, and *p* < 0.05 was considered statistically significant. Significance levels are indicated as follows: *p < 0.05; **p < 0.01; ***p < 0.001; ****p < 0.0001.

## Results

### Differential expression and prognostic analysis

**Figure 1:**
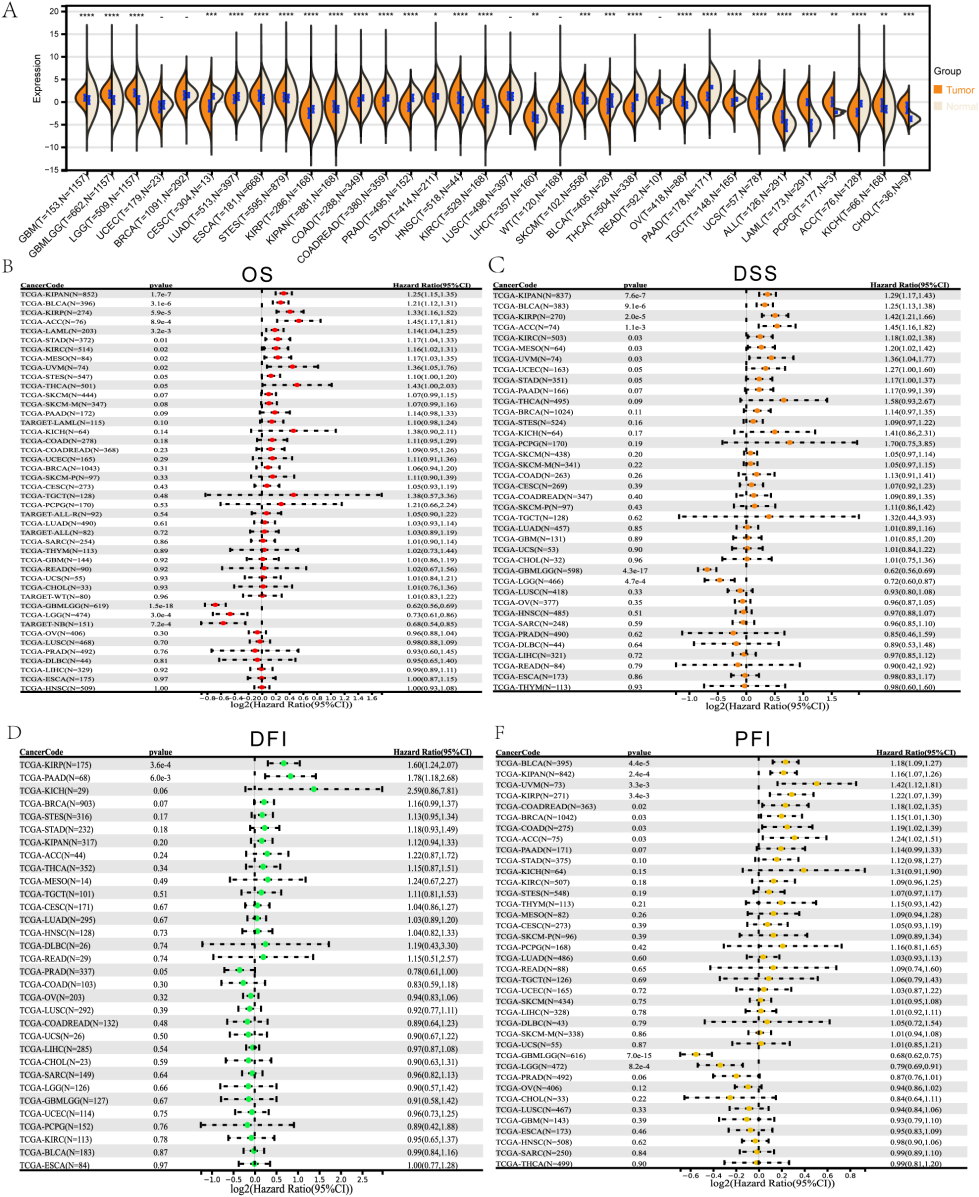
Identification of *ALDH1L2* expression and prognostic analysis. **(A)** Pan-cancer analysis of *ALDH1L2* for differential expression between tumor and normal tissues. **(B)** Pan-cancer analysis of *ALDH1L2* for Overall Survival (OS). **(C)** Pan-cancer analysis of *ALDH1L2* for Disease-Specific Survival (DSS). **(D)** Pan-cancer analysis of *ALDH1L2* for Disease-Free Interval (DFI). **(E)** Pan-cancer analysis of *ALDH1L2* for Progression-Free Interval (PFI). Overall Survival, OS; Disease-Specific Survival, DSS; Progression-Free Interval, PFI; Disease-Free Interval, DFI.

Compared with matched normal tissues, *ALDH1L2* mRNA levels were significantly dysregulated in multiple TCGA cancer types, with clear upregulation in a subset of tumors and downregulation in others (**Figure 1A**). Among urologic malignancies, *ALDH1L2* expression was markedly higher in KIRC than in adjacent kidney tissue, whereas BLCA and PRAD showed reduced expression relative to their corresponding normal controls.

Survival analyses across the pan-cancer cohort demonstrated that elevated *ALDH1L2* expression was associated with unfavorable overall survival (OS) in several entities, including KIPAN, BLCA, KIRP, adrenocortical carcinoma (ACC), KIRC, acute myeloid leukemia (LAML), and stomach adenocarcinoma (STAD), whereas low *ALDH1L2* expression predicted poor OS in some brain tumors (**Figure 1B**). Consistent patterns were observed for disease-specific survival (DSS), where high *ALDH1L2* expression indicated worse DSS in KIPAN, BLCA, KIRP, ACC, KIRC, LAML, and STAD, while reduced expression correlated with poorer DSS in glioma-related cohorts (**Figure 1C**). For DFI and PFI, *ALDH1L2* also showed tumor-type-dependent prognostic value: high expression was linked to shorter DFI in pancreatic adenocarcinoma (PAAD) and KIRP and to inferior PFI in BLCA, KIPAN, ACC, uveal melanoma (UVM), breast cancer (BRCA), colon adenocarcinoma (COAD), colorectal cancer (COADREAD), and KIRP, whereas low *ALDH1L2* expression was associated with unfavorable PFI in certain glioma subgroups (**Figures 1D, E**).

Clinically, *ALDH1L2* levels were significantly correlated with sex, TNM stage, pathological grade, and overall clinical stage in multiple tumor types, including urologic cancers (**Supplementary Figures S1B–G**). For example, *ALDH1L2* expression was positively related to T stage and higher pathological grade in KIRC and KIPAN, while age-stratified analyses showed a positive correlation between *ALDH1L2* and age in BLCA but negative correlations in KIPAN, KIRC, and PRAD, suggesting that *ALDH1L2* expression patterns may be influenced by both tumor biology and host factors(**Supplementary Figures S1B–G**).

**Figure 2:**
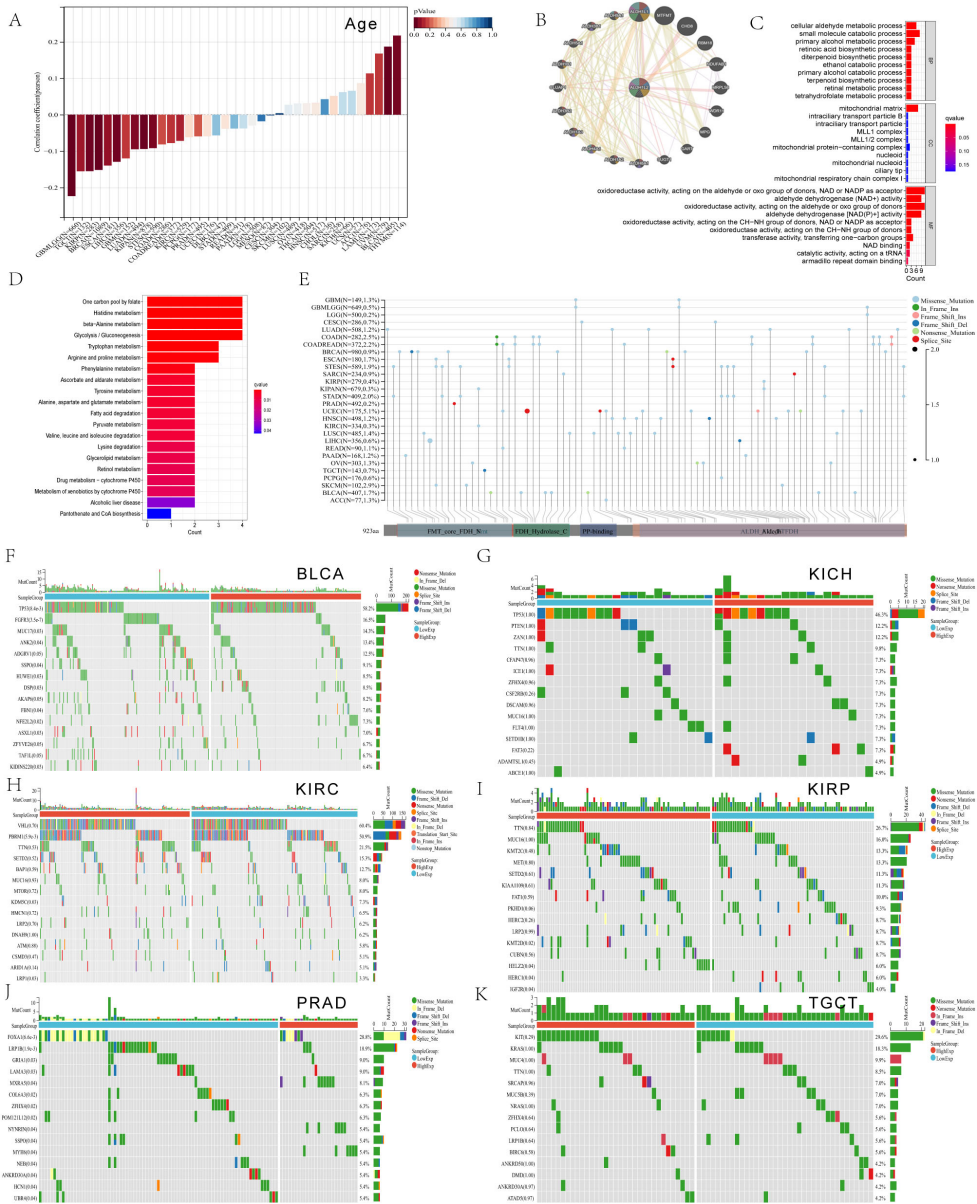
Analysis of *ALDH1L2* in relation to age, biological function, and mutational landscape. **(A)** Pan-cancer analysis of *ALDH1L2* expression by age. **(B)** Correlation analysis between *ALDH1L2* and the ALDH family as well as common pathogenic gene mutations. **(C)** Gene Ontology (GO) terms enriched among genes associated with *ALDH1L2*. **(D)** Kyoto Encyclopedia of Genes and Genomes (KEGG) pathways significantly enriched for *ALDH1L2*-related genes. **(E–H)** Somatic mutation profiles after stratifying patients by the median *ALDH1L2* expression: the 15 most frequently mutated genes are shown for **(E)** BLCA, **(F)** KIRC, **(G)** KIRP, and **(H)** PRAD. Key mutated genes include TP53, FGFR3, and MUC17 in BLCA; PBRM1 and KDM5C in KIRC; KMT2D and IGF2R in KIRP; and FOXA1 and LRP1B in PRAD.

The cancers consistently identified through both differential expression and prognostic analyses were KIPAN and BLCA for DSS and PFI. *ALDH1L2* expression also varied significantly across age groups (**Figure 2A**), correlating positively in BLCA and negatively in KIPAN, KIRC, and PRAD.

### Tumor heterogeneity, stemness, mutational landscape, RNA modifications, and immune checkpoint genes

**Figure 3:**
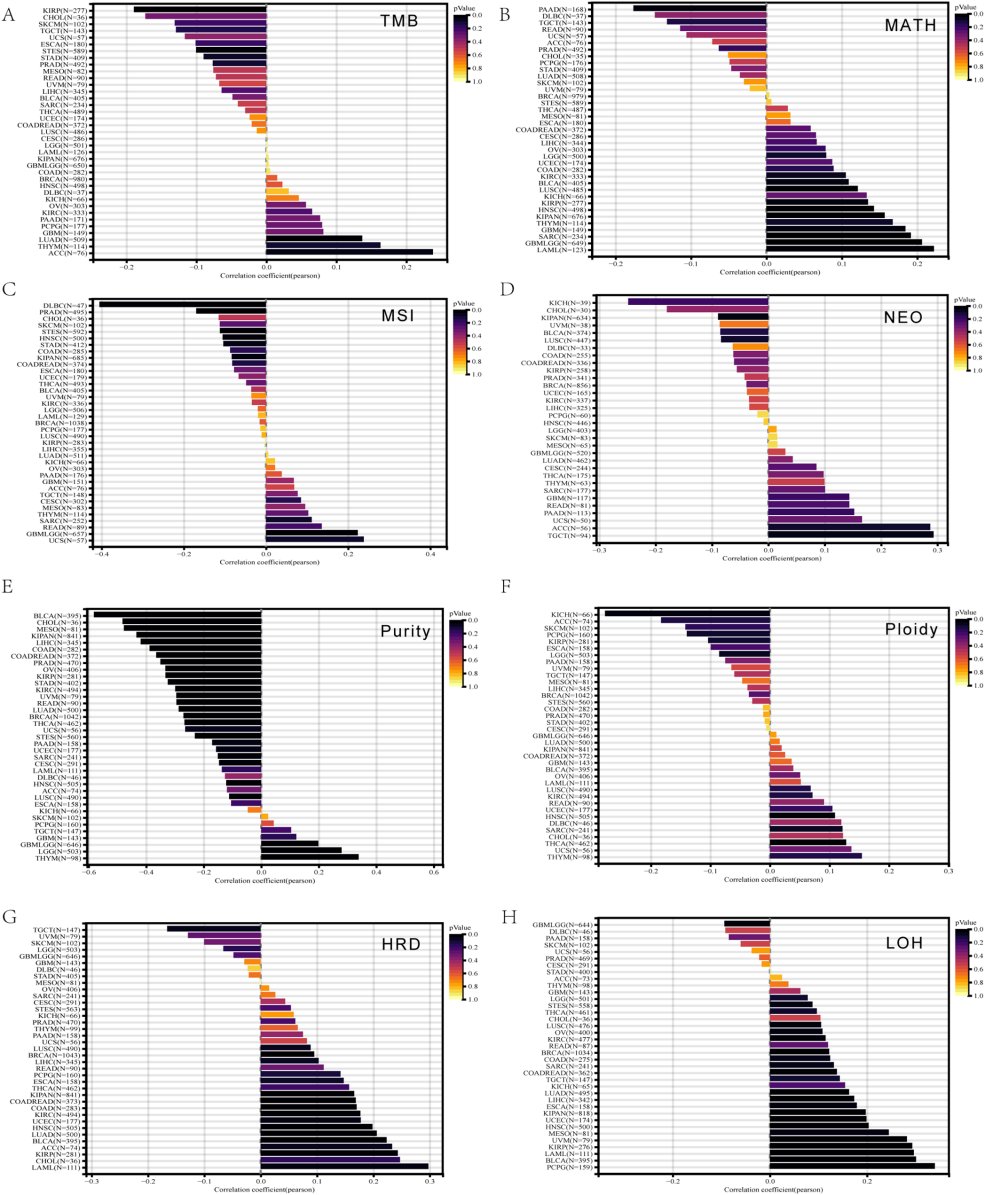
Pan-cancer spearman analysis of tumor heterogeneity and *ALDH1L2* expression. **(A)** Correlation between TMB and *ALDH1L2* levels. **(B)** Correlation between MATH and *ALDH1L2* levels. **(C)** Correlation between MSI and *ALDH1L2* levels. **(D)** Correlation between NEO load and *ALDH1L2* levels. **(E)** Correlation between Tumor Purity and *ALDH1L2* levels. **(F)** Correlation between Ploidy and *ALDH1L2* levels. **(G)** Correlation between HRD and *ALDH1L2* levels. **(H)** Correlation between LOH and *ALDH1L2* levels. TMB, tumor mutational burden; MATH, mutant-allele tumor heterogeneity; MSI, microsatellite instability; NEO, neoantigen; HRD, homologous recombination deficiency; LOH, loss of heterozygosity.

Regarding tumor heterogeneity, in PRAD, the mRNA expression of *ALDH1L2* was negatively correlated with MSI (R = -0.17) and tumor purity (R = -0.35) (**Figures 3A–H**). In BLCA, *ALDH1L2* mRNA expression showed positive correlations with HRD (R = 0.22) and LOH (R = 0.30), but was negatively correlated with tumor purity (R = -0.58) (**Figures 3A–H**). For TGCT, *ALDH1L2* mRNA levels showed an inverse association with HRD (R = −0.17). Within the pan-kidney cohort (KIPAN), higher *ALDH1L2* expression aligned with increased MATH (R = 0.15), HRD (R = 0.17), and LOH (R = 0.20), while it tracked with lower tumor purity (R = −0.43), reduced neoantigen load (NEO; R = −0.09), and decreased MSI (R = −0.08).

### Spearman analyses further linked *ALDH1L2* to tumor stemness in a cancer-type-specific manner

**Figure 4:**
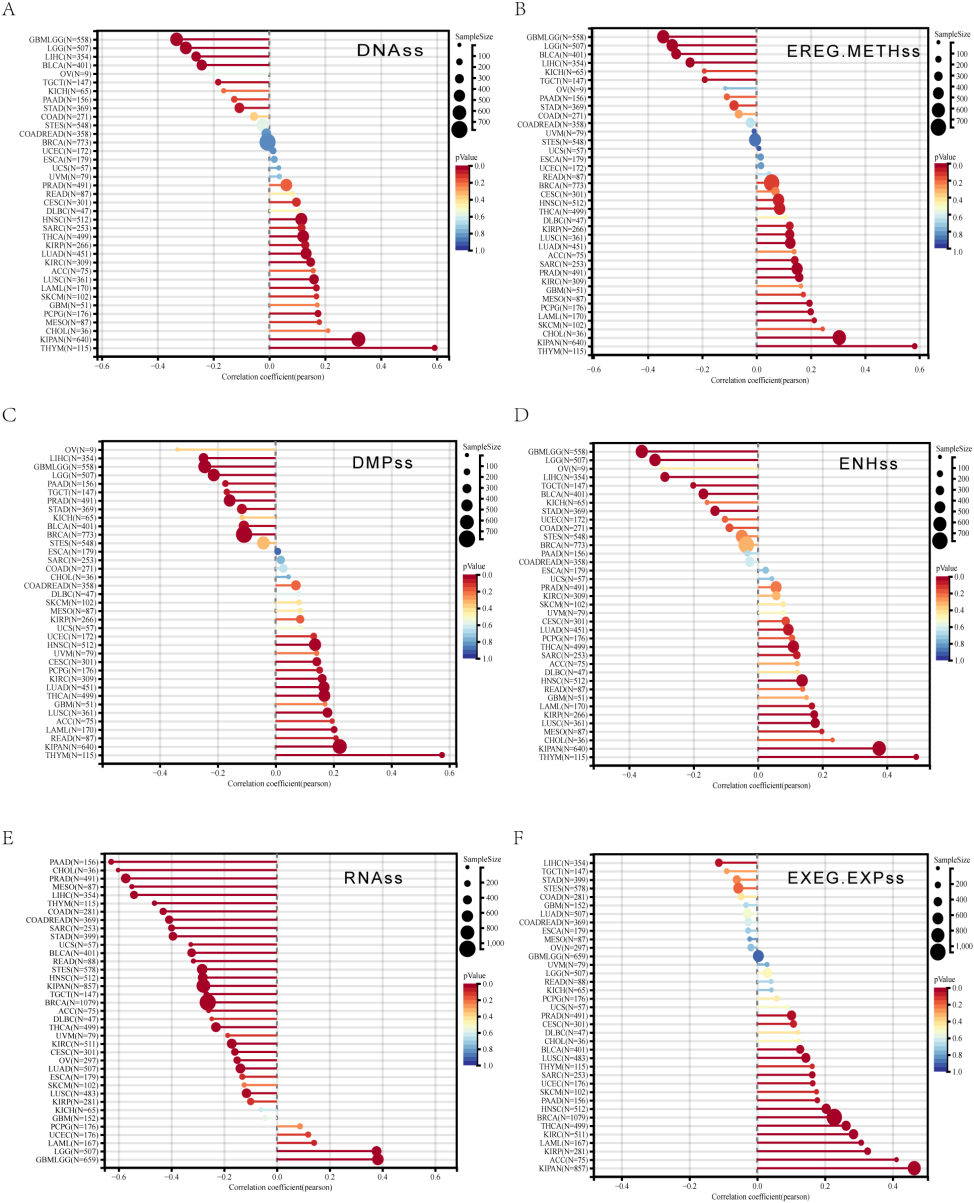
Pan-cancer spearman analysis of tumor stemness and *ALDH1L2* expression. **(A)** Correlation between DNAss and *ALDH1L2* levels. **(B)** Correlation between EREG-METHss and *ALDH1L2* levels. **(C)** Correlation between DMPss and *ALDH1L2* levels. **(D)** Correlation between ENHss and *ALDH1L2* levels. **(E)** Correlation RNAss and *ALDH1L2* levels. **(F)** Correlation between EREG-METHss and *ALDH1L2* levels.

In KIRC and KIPAN, *ALDH1L2* was positively associated with DNAss, EREG-METHss, DMPss, and EREG.EXPss, but showed an opposite trend for RNAss (**Figures 4A–F**).

In BLCA, *ALDH1L2* displayed concordant positive relationships with DNAss, EREG-METHss, DMPss, and ENHss (**Figures 4A–F**). By contrast, PRAD exhibited a mixed pattern: *ALDH1L2* was negatively related to RNAss and EREG.EXPss, yet remained positively associated with EREG-METHss (**Figures 4A–F**).

### Age, biological function, and genetic mutation landscape

The correlation of *ALDH1L2* with the ALDH family and commonly mutated genes is presented in **Figure 2B**. Functional annotation of *ALDH1L2*-associated genes was then performed using GO and KEGG analyses. GO terms highlighted enrichment in biological processes (BP) and molecular functions (MF), whereas KEGG results mainly pointed to pathways involved in one-carbon unit metabolism in folate-related reactions and amino-acid metabolic programs (**Figures 2C, D**).

The somatic alteration landscape of *ALDH1L2* is presented in **Figure 2E**. For downstream comparisons, patients were stratified into *ALDH1L2*-high and *ALDH1L2*-low groups according to the median expression within each tumor type. In BLCA, mutation frequencies differed between expression strata for TP53, FGFR3, MUC17, ANK2, SSPO, HUWE1, DSP, FBN1, NFE2L2, ASXL1, and KIDINS220 (**Figure 2F**). In KIRC, group-wise differences were most notable for PBRM1, KDM5C, and LRP1 (**Figure 2H**). In KIRP, the *ALDH1L2*-defined strata showed distinct mutation patterns involving KMT2D, HELZ2, HERC1, and IGF2R (**Figure 2I**). In PRAD, differential mutation prevalence was observed for FOXA1, LRP1B, GRIA1, LAMA3, MXRA5, COL6A3, ZFHX4, POM121L12, NYNRIN, SSPO, MYH6, NEB, ANKRD30A, HCN1, and UBR4 (**Figure 2J**). By contrast, no clear mutation-frequency differences were detected between *ALDH1L2*-high and -low groups in KICH (**Figure 2G**) or TGCT (**Figure 2K**).

### RNA modification-associated genes, immune checkpoints, and immunomodulatory genes

**Figure 5:**
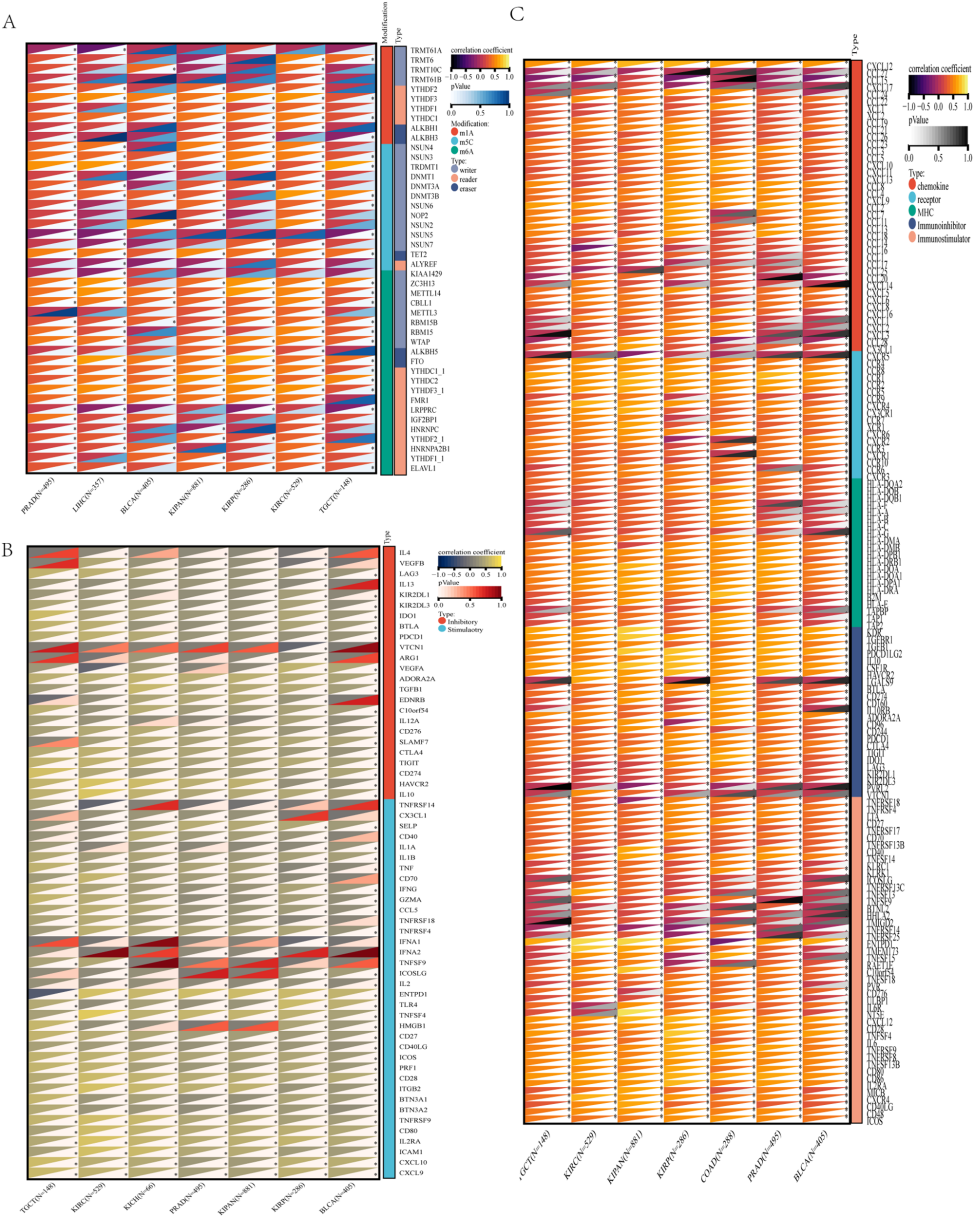
Relationships between *ALDH1L2* and RNA modification machinery, immune checkpoints, and immunoregulatory factors. Spearman’s rank correlation was used to evaluate the association between *ALDH1L2* expression and: **(A)** genes involved in RNA modifications, **(B)** immune checkpoint molecules, and **(C)** immunomodulatory genes.

Regarding RNA modifications, we found that the expression level of *ALDH1L2* in KICH, PRAD, BLCA, KIPAN, KIRP, KIRC, and TGCT was correlated with writer, reader, and eraser genes involved in m1A, m5C, and m6A RNA modifications. Multiple immune checkpoint genes (**Figure 5B**) and immunomodulatory genes (**Figure 5C**) showed associations with *ALDH1L2* expression levels across all seven cancer types.

### Multi-level analyses link *ALDH1L2* to the tumor microenvironment, validated by TISCH2 and TIMER3

**Figure 6:**
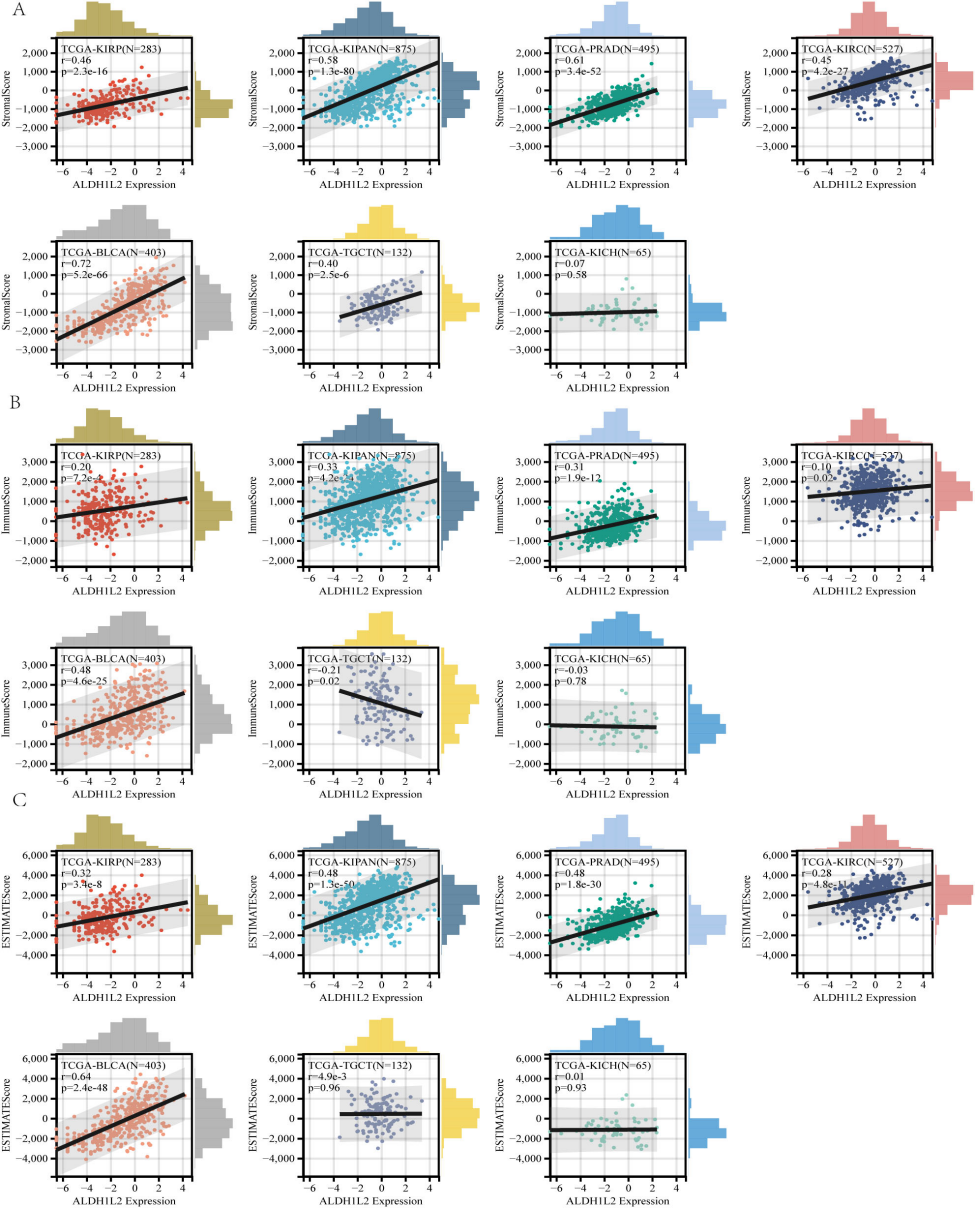
Association between *ALDH1L2* expression and tumor immune microenvironment. **(A)** Correlation between *ALDH1L2* expression and Stromal Score. **(B)** Correlation between *ALDH1L2* expression and Immune Score. **(C)** Correlation between *ALDH1L2* expression and ESTIMATE Score.

Across urologic and related tumor types, *ALDH1L2* expression displayed distinct microenvironmental associations as quantified by ESTIMATE (**Figures 6A–C**). In TGCT, *ALDH1L2* was aligned with a higher stromal component (stromal score, R = 0.40) but a reduced immune component (immune score, R = −0.21). In KIRP, increasing *ALDH1L2* levels tracked with higher stromal, immune, and composite ESTIMATE scores (R = 0.46, 0.20, and 0.32, respectively). Similar concordant patterns were observed in KIPAN (stromal, immune, and ESTIMATE scores: R = 0.58, 0.33, and 0.48) and PRAD (R = 0.61, 0.31, and 0.48). In KIRC, *ALDH1L2* showed more modest but still positive relationships with stromal, immune, and ESTIMATE scores (R = 0.45, 0.10, and 0.28), whereas BLCA exhibited comparatively stronger concordance across these metrics (R = 0.72, 0.48, and 0.64).

Immune deconvolution further indicated that *ALDH1L2* expression generally increased with immune cell infiltration (**Supplementary Figure S1A**). In PRAD, KIRP, KIPAN, and KIRC, higher *ALDH1L2* was associated with greater inferred abundance of B cells, CD4^+^ T cells, CD8^+^ T cells, neutrophils, macrophages, and dendritic cells. KICH showed positive associations limited to B cells, CD8^+^ T cells, macrophages, and dendritic cells. In BLCA, *ALDH1L2* correlated with CD4^+^ T cells, CD8^+^ T cells, neutrophils, macrophages, and dendritic cells. To strengthen the immune-related findings derived from bulk TCGA analyses, we performed external validation using single-cell and purity-adjusted resources. In the TISCH2 single-cell atlas (**Supplementary Figure S2A**), *ALDH1L2* expression was generally low across major lymphocyte compartments, whereas detectable signals were preferentially observed in stromal/mesenchymal subsets (e.g., fibroblasts and myofibroblasts) and plasma cell populations in several BLCA/PRAD/kidney cancer datasets, supporting a microenvironment-associated expression pattern. In parallel, TIMER3 analyses demonstrated that the correlations between *ALDH1L2* expression and immune infiltration estimates persisted after tumor purity adjustment, with macrophage-related signatures showing broadly positive associations across multiple deconvolution algorithms (**Supplementary Figure S2B**). Notably, the association with CD8^+^ T-cell–related signatures appeared more context-dependent across tumor types, consistent with the heterogeneous immune correlations observed in our pan-cancer TME analyses.

### *ALDH1L2* knockdown was verified and regulated migration and invasion in bladder, prostate, and renal cancer cells

**Figure 7:**
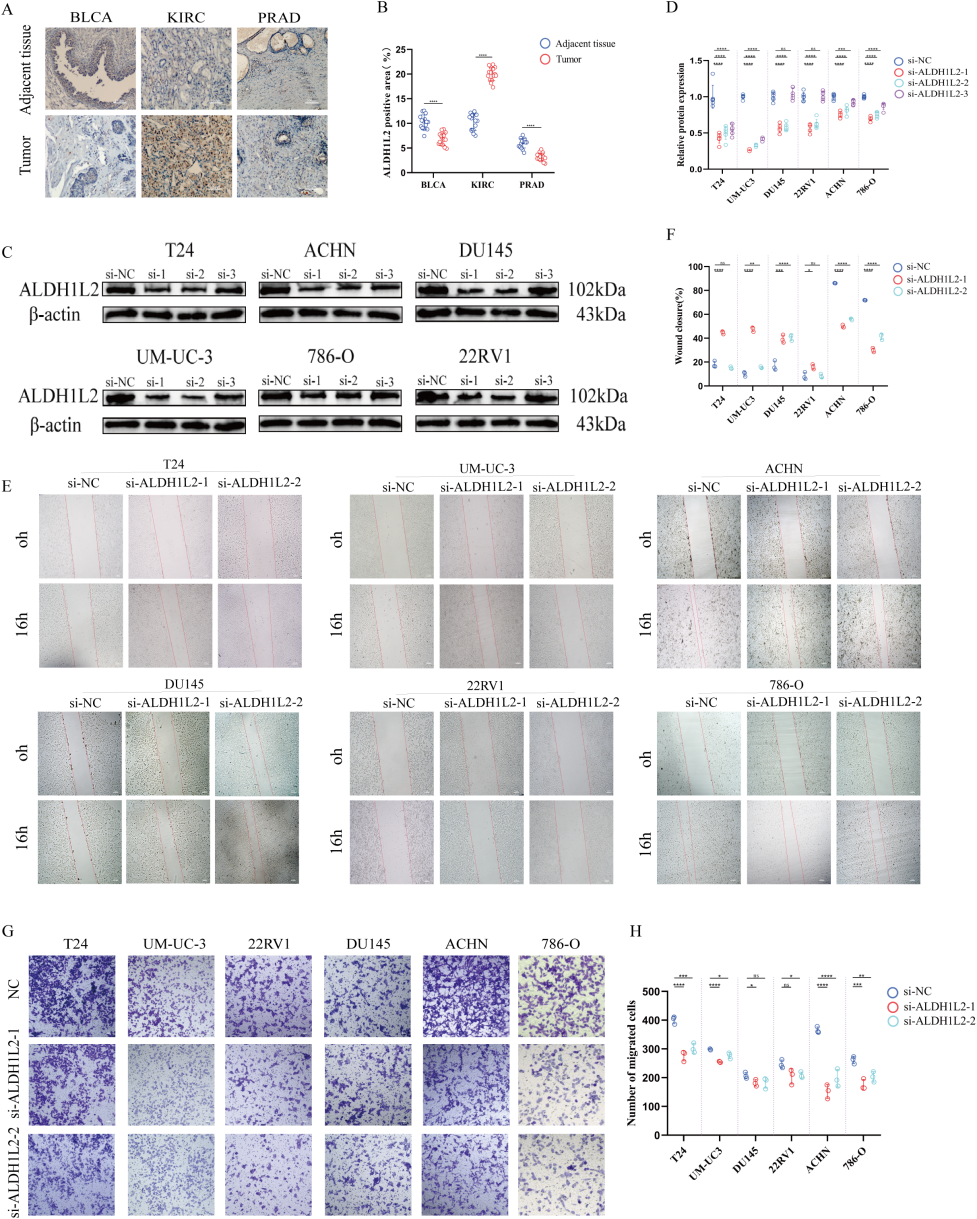
ALDH1L2 shows distinct expression patterns in urological cancers and differentially regulates cell migration and invasion. **(A)** Representative immunohistochemistry (IHC) images of ALDH1L2 in bladder urothelial carcinoma (BLCA), kidney renal clear cell carcinoma (KIRC), and prostate adenocarcinoma (PRAD) tissues and matched adjacent normal tissues (n = 15 paired samples per cancer type). **(B)** Quantification of ALDH1L2 staining in **(A)** using ALDH1L2-positive protein area (%). **(C)** Western blot validation of ALDH1L2 knockdown efficiency using three siRNAs (si-1, si-2, si-3) in BLCA (T24, UM-UC-3), KIRC (ACHN, 786-O), and PRAD (DU145, 22RV1) cell lines; β-actin served as the loading control. **(D)** Densitometric quantification of *ALDH1L2* protein levels in **(C)**, normalized to β-actin and expressed relative to the negative control (si-NC). **(E)** Representative images of wound-healing assays at 0 h and 16 h after scratching in the indicated cell lines transfected with si-NC or two selected siRNAs (si-*ALDH1L2–*1 and si-*ALDH1L2*-2). **(F)** Quantification of wound closure in **(E)**, calculated based on wound area measurements. **(G)** Representative images of Transwell migration assays following *ALDH1L2* knockdown in the indicated cell lines. **(H)** Quantification of migrated cells in **(G)**, presented as the number of migrated cells per field. (*p<0.05; **p<0.01; ***p<0.001; ****p<0.0001)

We found that the expression of *ALDH1L2* was slightly lower in BLCA compared to normal bladder tissue; its expression was significantly higher in KIRC than in normal kidney tissue; while in PRAD, *ALDH1L2* expression was also slightly lower than in normal prostate tissue (**Figures 7A, B**). The interference efficiency of siRNA targeting *ALDH1L2* was validated by qPCR and WB. Subsequently, si-*ALDH1L2–*1 and si-*ALDH1L2–*2 were selected for further experiments. Wound healing and Transwell assays were performed using two bladder cancer cell lines (T24 and UMUC3), two renal cancer cell lines (ACHN and 786-O), and two prostate cancer cell lines (22RV1 and DU145). The results indicated that si-*ALDH1L2* reduced the migratory ability of ACHN and 786-O cells but exhibited a certain enhancing effect on the migration of bladder and prostate cancer cells (**Figures 7C–H**).

### *ALDH1L2* knockdown enhances ROS generation, suppresses the Akt/mTOR/S6K signaling pathway, and inhibits the proliferation of renal carcinoma cells

**Figure 8:**
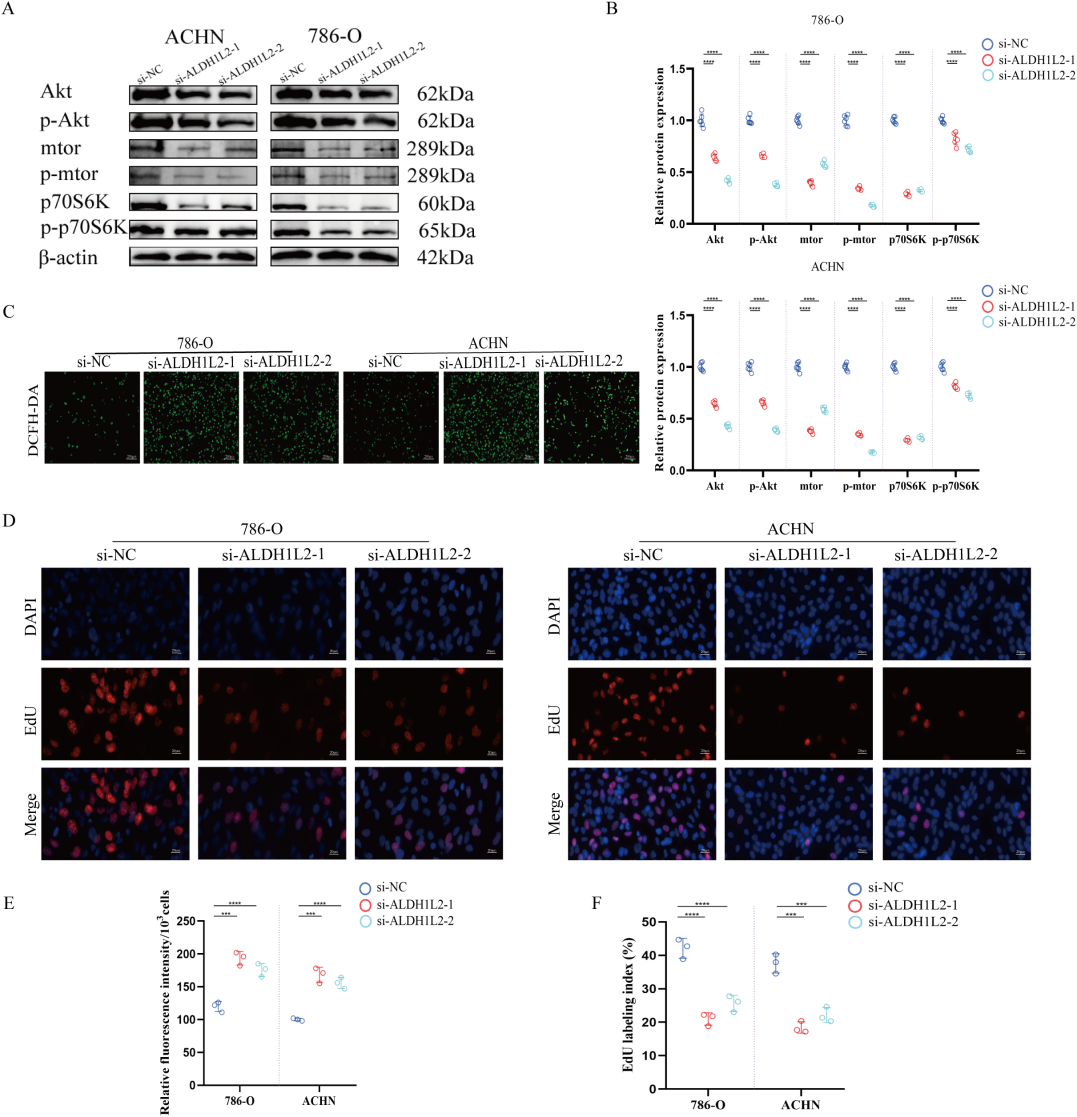
*ALDH1L2* knockdown suppresses the Akt/mTOR/S6K signaling pathway, enhances ROS generation, and inhibits renal cancer cell proliferation. **(A, B)***ALDH1L2* knockdown in renal cancer cells suppresses the Akt/mTOR/S6K signaling pathway and quantification results. **(C)** Representative fluorescence images of intracellular reactive oxygen species (ROS) detected by DCFH-DA staining in ACHN and 786-O cells transfected with si-NC or si-*ALDH1L2*. **(D)** Representative images of EdU incorporation assays in ACHN and 786-O cells following *ALDH1L2* knockdown; nuclei were counterstained with DAPI. **(E)** Quantification of ROS levels corresponding to **(C)**, expressed as relative fluorescence intensity (RFI) per 10³ cells. **(F)** Quantification of proliferative activity corresponding to **(D)**, presented as the EdU labeling index (%) = EdU^+^ nuclei/total DAPI nuclei × 100. (***p<0.001; ****p<0.0001)

Western blot analysis revealed that the activity of the Akt/mTOR/S6K signaling pathway was markedly suppressed following *ALDH1L2* knockdown (**Figures 8A, B**). Since *ALDH1L2* showed a good prognostic correlation and a consistent tumor-promoting phenotype in KIRC, we prioritized the kidney cancer model to explore the mechanistic nature of the ROS–Akt/mTOR axis, while ACHN and 786-O were more classic in the cell model of kidney cancer. We performed ROS generation assays in two renal carcinoma cell lines, ACHN and 786-O. The results demonstrated that knockdown of *ALDH1L2* significantly promoted ROS production (**Figures 8D, F**). Furthermore, 5-ethynyl-2’-deoxyuridine (EdU) incorporation assays indicated that *ALDH1L2* knockdown effectively inhibited the proliferation of both ACHN and 786-O cells (**Figures 8C, E**).

## Discussion

This study represents the first systematic effort to elucidate the expression patterns and biological roles of *ALDH1L2* across multiple cancer types by integrating pan-cancer analysis, immunohistochemical validation, and functional experiments. Pan-cancer analysis revealed that *ALDH1L2* is differentially expressed in clear cell renal cell carcinoma (KIRC), bladder urothelial carcinoma (BLCA), and prostate adenocarcinoma (PRAD), with its expression levels significantly correlating with patient prognosis. Immunohistochemical staining further confirmed the downregulation of *ALDH1L2* expression at the tissue level. Notably, functional experiments demonstrated that *ALDH1L2* knock down exerted opposing effects depending on the cancer type: it suppressed cell migration and proliferation in KIRC, whereas it promoted these processes in BLCA and PRAD. These findings indicate that *ALDH1L2* can play a context-dependent, dual role in tumorigenesis, which is shaped by tissue origin and metabolic background.

Previous studies have established that *ALDH1L2* plays a critical role in mitochondrial one-carbon metabolism, NADPH production, and lipid homeostasis (4, 5, 7, 28). Loss of *ALDH1L2* function leads to reduced antioxidant capacity, metabolic reprogramming, and the subsequent accumulation of lipids and ROS (29, 30). In hormone-dependent cancers such as breast and prostate cancer, the excessive utilization of lipids and cholesterol is a key driver of cell proliferation (31–36), which may explain the tumor-promoting effect of *ALDH1L2* observed in BLCA and PRAD. Cellular senescence represents a context-dependent program that can restrain proliferation yet also remodel the tumor ecosystem, thereby shaping therapy responsiveness and disease evolution (37). KIRC cells exhibit high dependence on mitochondrial metabolism; therefore, *ALDH1L2* deficiency is more likely to disrupt NADPH balance, induce ROS accumulation, and activate the Akt/mTOR pathway, thereby enhancing proliferative capacity. Such cancer type-specific effects underscore the complexity of *ALDH1L2* function and untangle its unique position at the intersection of metabolic and signaling networks.

There is now uncertainty in the treatment of the tumor microenvironment, as immune infiltration does not necessarily translate into effective anti-tumor immunity, as bone marrow-driven immunosuppression and CD8+ T cell depletion can decouple immune abundance from immune capacity (38). And advancements in tumor immunotherapy targeting one-carbon metabolism have demonstrated significant progress. This metabolic network influences immunotherapy outcomes via several interconnected pathways, notably the folate cycle, the methionine cycle, and the transsulfuration pathway (39, 40). One-carbon metabolism plays an important role in circadian rhythms, which can influence the tumor microenvironment by affecting immune components, metabolic status, and treatment vulnerability (41). Consequently, key enzymes within one-carbon metabolism—including serine hydroxymethyltransferase (SHMT), methylenetetrahydrofolate dehydrogenase (MTHFD), thymidylate synthase (TYMS), and dihydrofolate reductase (DHFR)—have become a major focus of investigation. Inhibitors of SHMT have been shown to suppress the growth of pancreatic tumor xenografts and demonstrate anticancer activity *in vivo (*42). They also exert potent antitumor effects through mechanisms such as inducing G1–S cell cycle arrest and inhibiting breast cancer growth (43). Several MTHFD inhibitors have exhibited favorable *in vivo* antitumor activity following oral administration (44, 45). Meanwhile, TS and DHF reductase inhibitors have already been applied in clinical settings (46–49), with multiple compounds demonstrating substantial antitumor effects in clinical trials (50, 51). The regulated cell death process is closely linked to redox homeostasis and anti-cancer immunity, supporting the translational theory for ROS-related vulnerability combined with existing signal inhibitors (52). Hypoxia is a common metabolic feature of solid tumors, which may weaken the activity of natural killer (NK) cells, thereby promoting immune escape (53). Emerging evidence suggests that microbiota–immune crosstalk may critically shape the urinary tumor immune milieu and influence disease behavior as well as treatment responses, highlighting potential confounders and opportunities for stratification (54). Therefore, targeting *ALDH1L2* holds promising therapeutic potential, although further exploration is required for the development of antitumor therapies based on one-carbon metabolism.

Mechanistic studies indicated that *ALDH1L2* knock down activates the Akt/mTOR pathway and promotes ROS generation. The Akt/mTOR signaling axis plays a central role in metabolic reprogramming and growth regulation, while moderate levels of ROS can act as signaling molecules to further drive tumor proliferation. EdU assay confirmed that *ALDH1L2* deletion enhances cell proliferative capacity. These results suggest the existence of an “*ALDH1L2*–NADPH–ROS–Akt/mTOR–Proliferation” functional axis, providing a new perspective for understanding the tumor-suppressive role of *ALDH1L2* in KIRC. Future studies utilizing Akt/mTOR inhibitors or ROS scavengers may help validate this causal relationship and offer novel strategies for the treatment of renal cell carcinoma.

Clinically, *ALDH1L2* expression holds potential as a prognostic biomarker for KIRC patients, demonstrating considerable predictive value when combined with immune infiltration and molecular subtyping analyses. To minimize the impact of tumor purity on our findings, we additionally analyzed these associations using TIMER2.0 alongside purity-adjustment methods, and the results remained consistent. In addition to host-intrinsic determinants, the intratumoral microbiota has been implicated in shaping immunotherapy responsiveness, suggesting that microenvironmental context may modulate biomarker–immune associations across cohorts (55). However, the specific mechanisms underlying its tumor-promoting role in BLCA and PRAD require further investigation, particularly regarding its relationship with cholesterol homeostasis, androgen signaling, and the tumor microenvironment. Against the backdrop of the increasing global burden of urothelial malignancies (16), accumulating evidence indicates intersections between age-related metabolic remodeling—such as hypoxia, myeloid suppression (8), and microbiota immune contextualization (38, 53–55)—and drug sensitivity governed by redox regulation (52). This provides a strong rationale for exploring *ALDH1L2* as a context-dependent metabolic−immune hub in urological tumors.

Despite providing multi-layered evidence, this study has several limitations. Our experimental validation was primarily conducted in ex vivo cellular models, lacking support from animal studies or clinical samples. Moreover, the direct causal relationship between *ALDH1L2* and immune−cell infiltration remains unclear. Systematic investigations into the expression differences of *ALDH1L2* across diverse populations are still lacking. Our detection methods were unable to distinguish between the potential catalytic effects of *ALDH1L2* and its non−enzymatic or structural functions. Although TCGA provides large−scale evidence, residual batch effects and cross−cohort clinical heterogeneity may influence the pan−cancer correlations. Future studies should focus on validating the proposed mechanisms in animal models, further dissecting the interactions between *ALDH1L2* and lipid metabolism or immune microenvironment factors, and evaluating its clinical feasibility as a diagnostic or therapeutic target.

In summary, this study uncovers the differential expression and clinical significance of *ALDH1L2* in various cancers, and confirms its heterogeneous roles in urological tumors through immunohistochemistry and functional experiments. Further mechanistic studies suggest that *ALDH1L2* influences KIRC cell proliferation by regulating the Akt/mTOR pathway and ROS dynamics. Collectively, these results position *ALDH1L2* as a candidate marker with potential translational relevance and motivate further studies on how mitochondrial one-carbon/redox metabolism interfaces with growth signaling in distinct tumor settings.

## Conclusion

In this work, we integrate pan-cancer profiling with tissue and cell-based validation to define *ALDH1L2* as a context-dependent regulator linking mitochondrial redox control to growth signaling in urologic malignancies. In KIRC, loss of *ALDH1L2* drives ROS accumulation and Akt/mTOR activation, thereby promoting cell proliferation and migration; conversely, it exhibits an opposing effect in BLCA and PRAD. These observations support the utility of *ALDH1L2* in molecular stratification and suggest that therapeutic strategies combining mTOR pathway inhibition with redox regulation may be worthy of evaluation in appropriately screened patient subgroups. Given that relevant therapeutic agents have already entered clinical use, future validation of *ALDH1L2*-based stratification and corresponding targeting strategies across different cancer types holds clear translational promise.

With the increasing burden of urinary tract malignancies worldwide [xx], more and more evidence highlights the convergence of age-related metabolic remodeling [xx], such as hypoxia, myeloid suppression and microbiota immune contextualization [xx–xx], and redox-modulated druggable susceptibility [xx], providing a strong basis for exploring *ALDH1L2* as context-dependent metabolic immune junctions in urologic tumors.

## Data Availability

The original contributions presented in the study are included in the article/**Supplementary Material**. Further inquiries can be directed to the corresponding authors.

## Supplementary material

Supplementary Figure 1 *ALDH1L2* correlation with immune cells and clinical characteristics. **(A)** Correlation *ALDH1L2* with immune cells, **(B–G)***ALDH1L2* Correlation with clinical characteristics such as gender, TNM stage, clinical stage, and clinical grade.

Supplementary Figure 2 Single-cell and tumor purity–adjusted validation of *ALDH1L2* immune associations. **(A)** TISCH2-based single-cell RNA-seq analysis showing the average expression of *ALDH1L2* across annotated cell types in curated BLCA, PRAD, and kidney cancer datasets. Color intensity indicates mean expression levels displayed as log(TPM/10 + 1). **(B)** TIMER3 analysis depicting tumor purity–adjusted partial correlations between *ALDH1L2* expression and estimated infiltration levels of macrophage subsets and CD8^+^ T-cell–related signatures across TCGA cohorts (BLCA, KICH, KIRC, KIRP, PRAD, and TGCT), calculated using multiple immune deconvolution algorithms.
