# Supplementary figures and images for: ALDH1L2 orchestrates redox–growth coupling in renal carcinoma: pan-cancer evidence and mechanistic validation of the ROS–Akt/mTOR/S6K axis
Source: Front Immunol. 2026 Feb 12;17:1768010. doi: 10.3389/fimmu.2026.1768010 (PMC12935911; doi:10.3389/fimmu.2026.1768010)

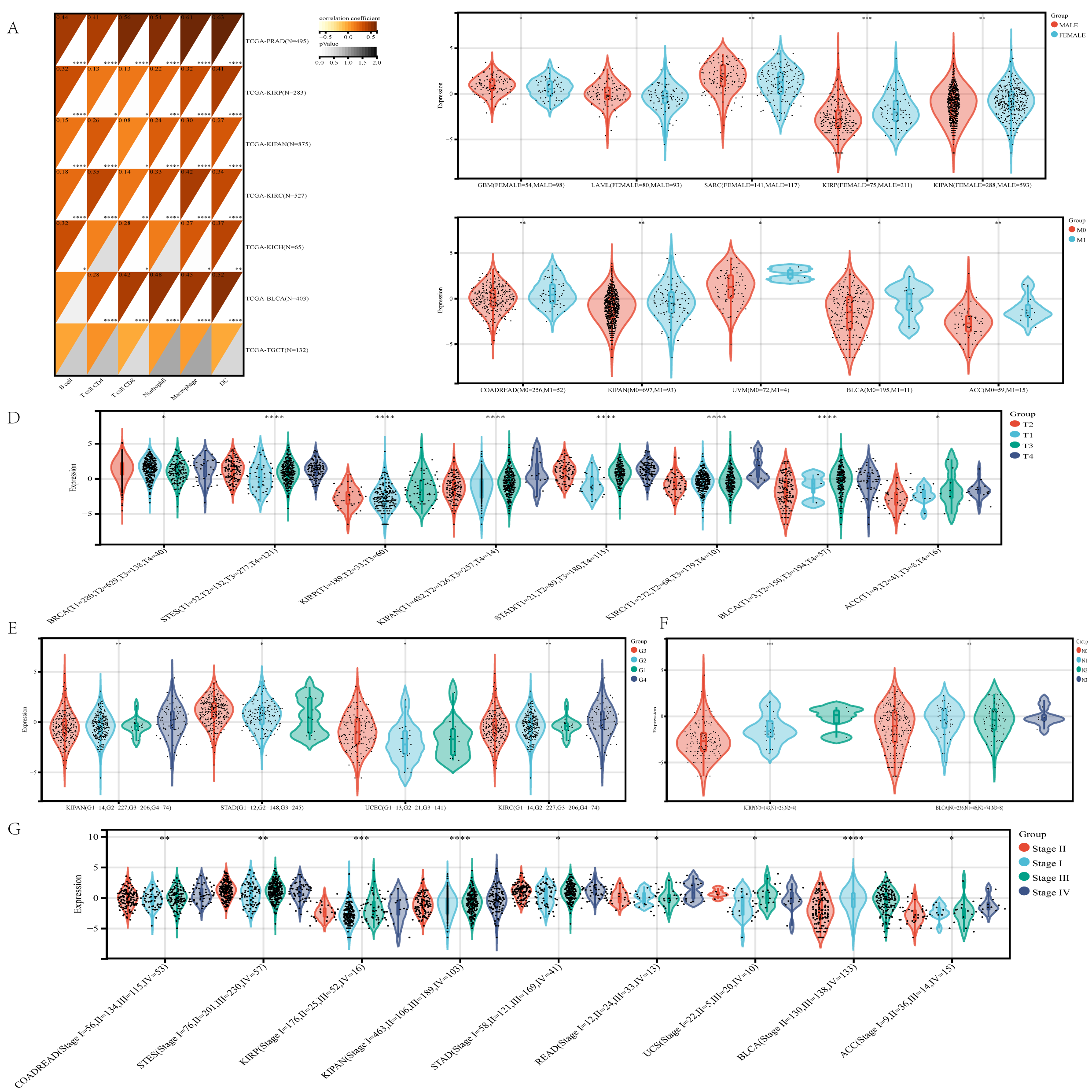

Supplement: Supplementary Figure 1 — ALDH1L2 correlation with immune cells and clinical characteristics. (A) Correlation ALDH1L2 with immune cells, (B–G)ALDH1L2 Correlation with clinical characteristics such as gender, TNM stage, clinical stage, and clinical grade. [file Image1.tif]

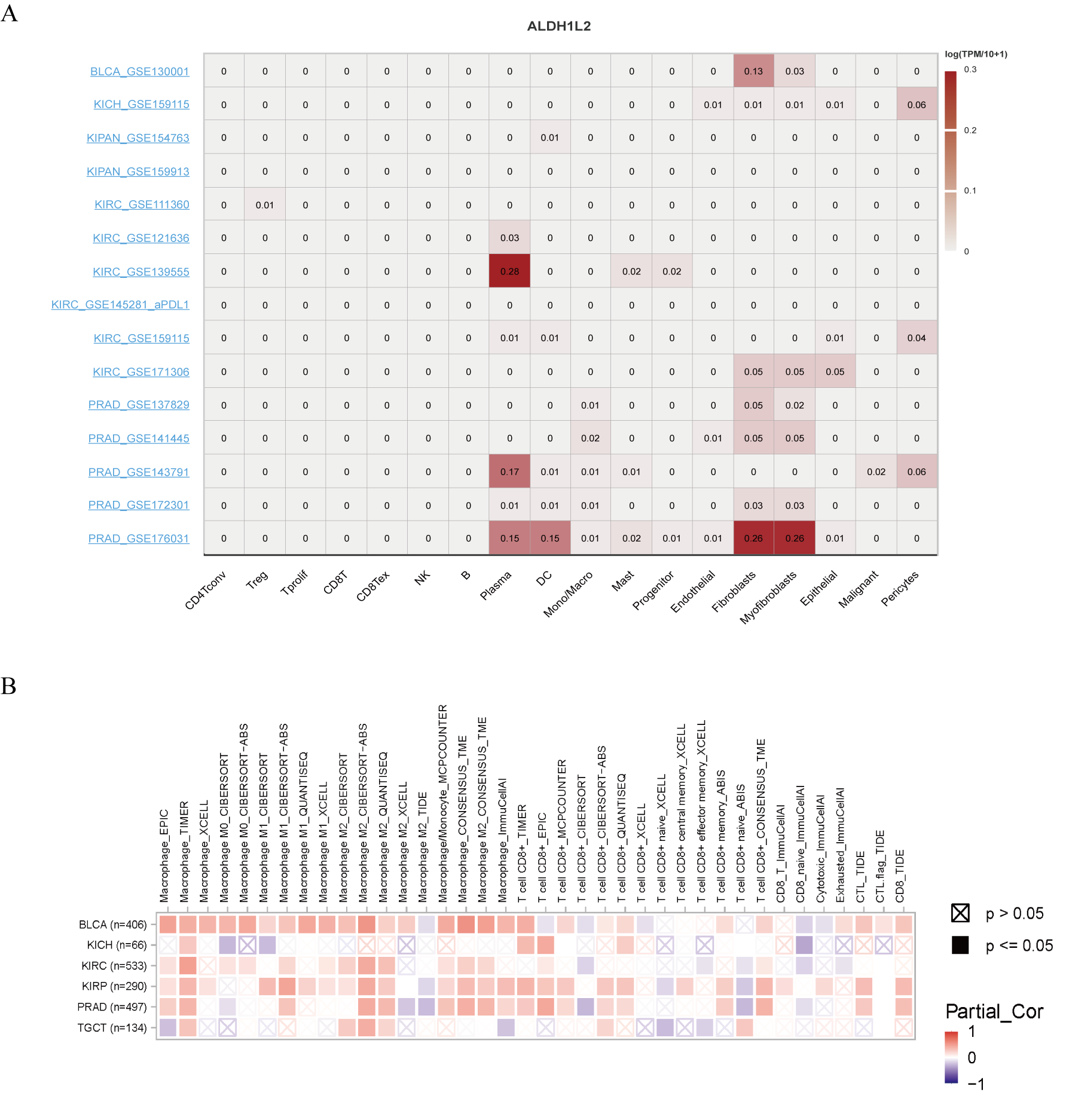

Supplement: Supplementary Figure 2 — Single-cell and tumor purity–adjusted validation of ALDH1L2 immune associations. (A) TISCH2-based single-cell RNA-seq analysis showing the average expression of ALDH1L2 across annotated cell types in curated BLCA, PRAD, and kidney cancer datasets. Color intensity indicates mean expression levels displayed as log(TPM/10 + 1). (B) TIMER3 analysis depicting tumor purity–adjusted partial correlations between ALDH1L2 expression and estimated infiltration levels of macrophage subsets and CD8+ T-cell–related signatures across TCGA cohorts (BLCA, KICH, KIRC, KIRP, PRAD, and TGCT), calculated using multiple immune deconvolution algorithms. [file Image2.tif]
